# A Systematic Review on Neurological Aspects of COVID-19: Exploring the Relationship Between COVID-19-Related Olfactory Dysfunction and Neuroinvasion

**DOI:** 10.3389/fneur.2022.887164

**Published:** 2022-07-15

**Authors:** Sujata Purja, SuA Oh, EunYoung Kim

**Affiliations:** ^1^Evidence-Based and Clinical Research Laboratory, Department of Health, Social and Clinical Pharmacy, College of Pharmacy, Chung-Ang University, Seoul, South Korea; ^2^The Graduate School for Food and Drug Administration, The Graduate School for Pharmaceutical Industry Management, College of Pharmacy, Chung-Ang University, Seoul, South Korea

**Keywords:** COVID-19, SARS-CoV-2, anosmia, cerebrospinal fluid, neuroinvasion

## Abstract

**Objectives:**

To identify neurological aspects of Coronavirus disease 2019 (COVID-19) and to investigate COVID-19 infected patients with and without olfactory dysfunction in relation to polymerase chain reaction (PCR) assay results for severe acute respiratory syndrome coronavirus-2 (SARS-CoV-2) in the cerebrospinal fluid (CSF).

**Methods:**

PubMed and EMBASE databases were searched until March 26, 2021, for observational studies with COVID-19 patients that had performed CSF PCR assay due to the neurologic symptom and reported anosmia status.

**Results:**

Initially, 2,387 studies were identified;167 studies performed SARS-CoV-2 CSF PCR assay, of which our review comprised 45 observational studies that conducted CSF PCR assay for SARS-CoV-2 in 101 patients and reported anosmia status in 55 of 101 patients. Central and peripheral neurological manifestations observed in COVID-19 patients were diverse. The most common neurological diagnoses were Guillain-Barré syndrome (GBS) and its variants (24%), followed by encephalopathy (21%). The SARS-CoV-2 PCR assay was positive in only four CSF samples, of which two patients had olfactory dysfunction while the others did not.

**Conclusions:**

The neurological spectrum of COVID-19 is diverse, and direct neuroinvasion of SARS-CoV-2 is rare. The neuroprotection against SARS-CoV-2 in COVID-19 patients with anosmia is controversial, as an equal number of patients with and without olfactory dysfunction had positive CSF PCR results for SARS-CoV-2 in our study, and further studies are required to provide more insight into this topic.

## Introduction

The olfactory nerve connects the nasal cavity to the central nervous system (CNS) and provides a neuroinvasive shortcut to respiratory neurotropic viruses (1). The detection of severe acute respiratory syndrome coronavirus-2 (SARS-CoV-2) in the olfactory nerve and CNS of patients with coronavirus disease 2019 (COVID-19) suggests that SARS-CoV-2 has neuroinvasive potential *via* the olfactory pathway (2). Although SARS-CoV-2 neuroinvasion is uncommon, CNS viral transmission poses a significant threat to life (3).

Previous animal studies have demonstrated that respiratory neurotropic viral invasion induces apoptosis of olfactory receptor neurons (ORNs), preventing the viral transmission to the olfactory bulb and the CNS (4, 5). Although the exact mechanism underlying COVID-19 related anosmia is unclear, human and animal studies have shown that anosmia is a consequence of a host defense mechanism against viral invasion involving the damage of olfactory epithelium might provide neuroprotection (2, 5–9). Furthermore, anosmia is frequently seen in milder forms of COVID-19 with a lower mortality rate (10, 11). Therefore, neuroprotection is anticipated in COVID-19 patients with anosmia.

Understanding the underlying mechanism and prognostic value of COVID-19-related anosmia will aid better patient management since olfactory dysfunction is often associated with several neurological disorders (12). This systematic review aimed to compile studies involving COVID-19 patients with neurological manifestations who have undergone polymerase chain reaction (PCR) testing for SARS-CoV-2 in cerebrospinal fluid (CSF) and reported the patient's anosmia status for identifying neurological aspects of COVID-19 and exploring the COVID-19 infected patients with or without anosmia in relation to their CSF PCR assay results.

## Methods

### Eligibility Criteria

The observational studies related to CSF analysis of COVID-19 patients with neurological symptoms were included. Target patients were COVID-19 patients diagnosed based on either positive SARS-CoV-2 PCR or serologic testing who had a neurological manifestation and have undergone SARS-CoV-2 CSF PCR testing to identify COVID-19-related neurological disorders. Studies that conducted CSF PCR assay for SARS-CoV-2 but did not report information on the status of anosmia were excluded. The study covered primary, retrievable scientific literature available in English. Collected data were each patient's sex and age distribution, SAR-CoV-2 CSF PCR assay, neurological presentation, treatment, and outcome. Therefore, studies that did not report these data properly were also excluded.

### Search Strategy

We conducted a broad literature search of databases such as EMBASE and PubMed until March 26, 2021, following preferred reporting items for systematic reviews and meta-analysis (PRISMA) checklist (13) for studies that performed CSF PCR assay for SARS-CoV-2 in COVID-19 patients using population search terms “SARS-CoV-2” or “COVID-19” and intervention search terms “brain” or “cerebrospinal fluid” or “anosmia”.

### Study Selection

Two independent authors screened studies based on the titles and abstracts. Any studies relevant to the CSF analysis of patients with COVID-19 were advanced to the second stage of the review. Full texts were reviewed using the eligibility criteria mentioned above in the second screening. Any disagreement between the authors was resolved by discussion.

### Risk of Bias Assessment

The Joanna Briggs Institute (JBI) critical appraisal checklist was used to assess the risk of bias in each included study (14).

### Data Extraction and Analysis

Two authors independently collected the data items included in the study design for each eligible study. For evaluating neurological aspects of COVID-19, individual patient data on neurological presentation, treatment, and outcomes were collected. The data items included individual's age and sex distribution, CSF PCR assay result, anosmia status, COVID-19-related neurological symptoms, neurological diagnosis, treatment, and outcomes. Each COVID-19 patient's data with neurological manifestations who had undergone CSF PCR testing for SARS-CoV-2 to identify COVID-19-related neurological disorders was summarized to characteristics, clinical presentation, SARS-CoV-2 PCR assay results, neurological diagnosis, treatment, and outcomes.

## Results

### Study Selection

**Figure 1:**
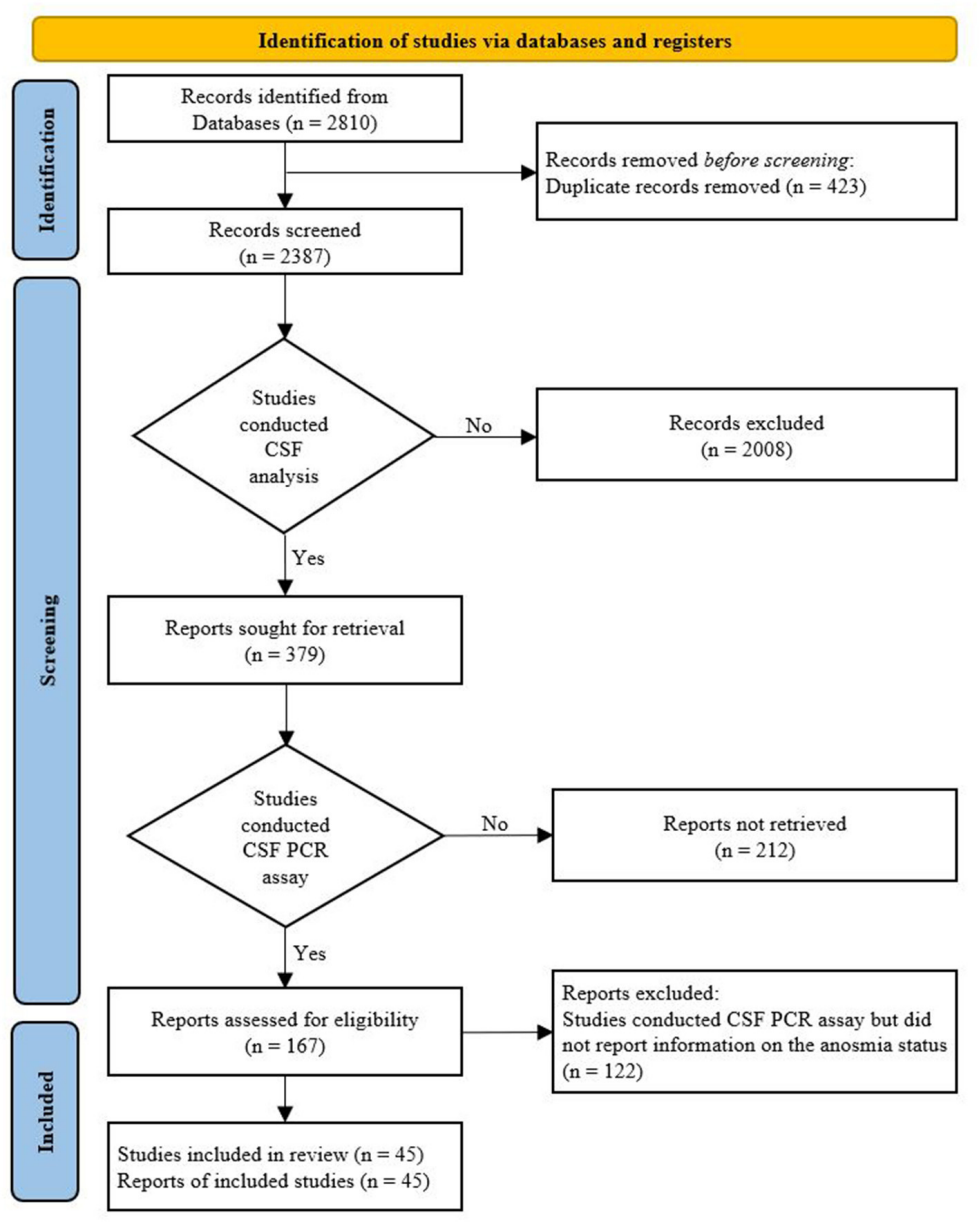
PRISMA flowchart.

In total, 2,387 studies were identified through a literature search after removing duplicates. After preliminary screening based on the titles and abstracts, a total of 379 studies related to CSF analysis of COVID-19 patients with neurological symptoms were included; among them, 167 studies (44%) that conducted PCR tests for SARS-CoV-2 in CSF were selected for full-text review. A total of 122 studies that conducted CSF PCR assay for SARS-CoV-2 but did not report information on the status of anosmia were excluded. Thus, only 45 articles that met the inclusion criteria were included in our study (15–59). A flow diagram of the study selection process is shown in Figure 1.

### Risk of Bias

Overall, the risk of bias in the included studies was low except for three studies (15, 21, 57). The summary of JBI critical appraisal results for case reports and case series can be seen in Supplementary Tables 1, 2.

### Participants and Characteristics of Studies

**Figure 2:**
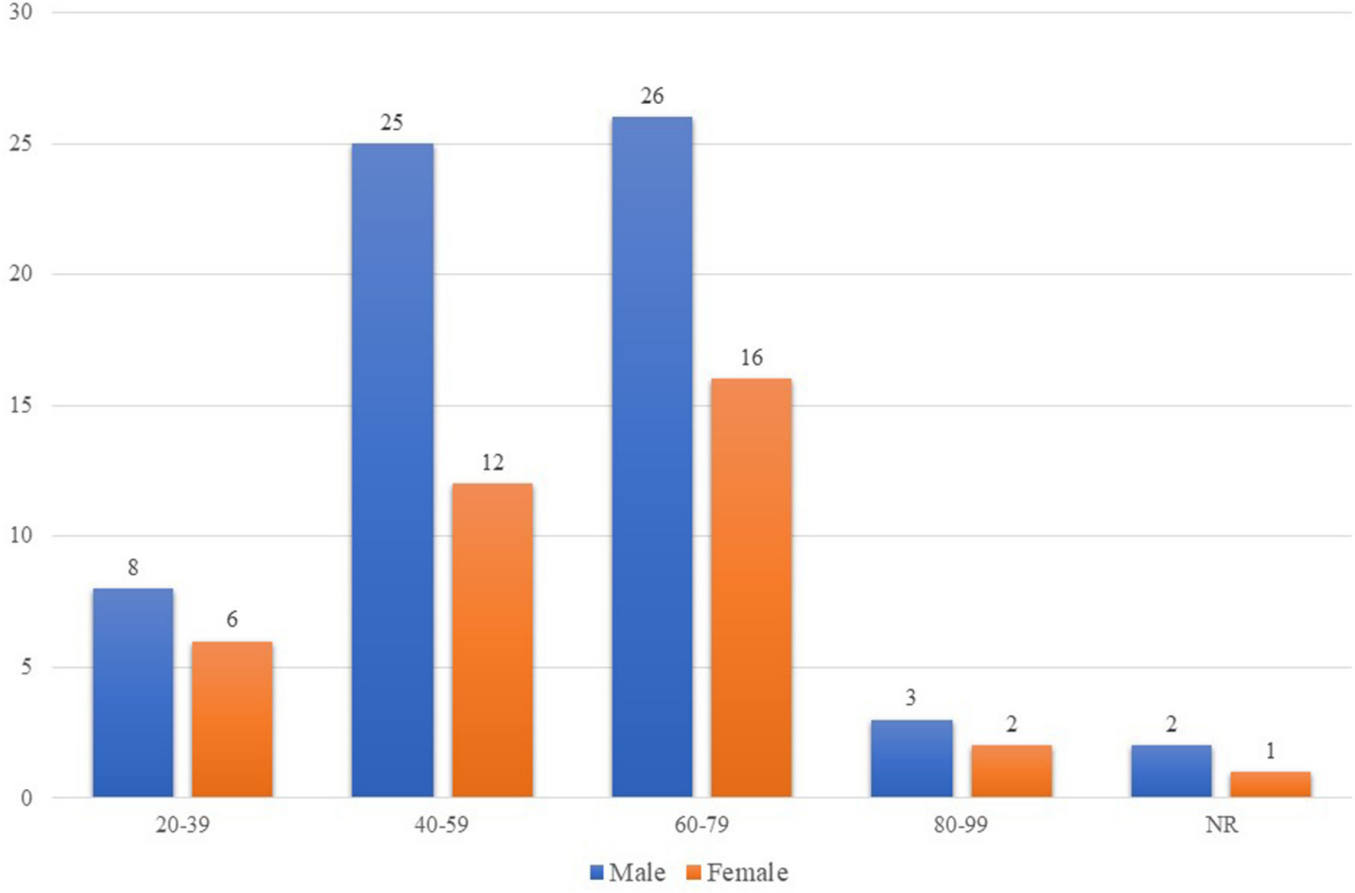
Age and sex distribution of COVID-19 patients who underwent CSF PCR assay for SARS-CoV-2. The number of men and women infected with COVID-19 who developed severe neurological manifestations and underwent CSF PCR assay for SARS-CoV-2 increased as the age of the individuals increased. The impact of COVID-19 was higher in patients aged 60-70 years old of both sexes. In addition, there were more COVID-19 infected men than COVID-19 infected women of all ages. COVID-19, coronavirus disease 2019; CSF, cerebrospinal fluid; PCR, polymerase chain reaction; SARS-CoV-2, severe acute respiratory syndrome coronavirus-2. NR denotes that the ages of two males and one female were not reported.

The total number of participants was 104, while the SARS-CoV-2 CSF PCR testing was performed on only 101 patients. Table 1 shows the characteristics of the 101 participants included in the review. More than 63.4% (64/101) were men. The mean age was 57 ± 16.37 years. The number of men and women infected with COVID-19 increased with age. COVID-19 infected patients of both sexes, predominantly in the 60–79 age group (Figure 2).

**Table 1:** Characteristics of studies included in the review.

**References**	**Total case**	**Age/sex**	**SARS-CoV-2 diagnostic**	**CSF PCR**	**LOS**	**Neurological diagnosis**	**Treatment**	**Outcome**
**Case report**								
Andriuta et al. (15)	2	^NR^/F	NPS	Neg	Yes	Encephalopathy	NR	NR
		^NR^/M	NS	Neg	NR	Encephalopathy	NR	Unawaken
Assini et al. (16)	2	55/M	OPS	Neg	Yes	Polyradicoloneuritis	Idrossichlorochine, arbidol, L/R, IVIG	Non-fatal
		60/M	NPS	Neg	NR	Polyradicoloneuritis	HCQ, ART, Tocilizumab, IVIG	Non-fatal
Atakla et al. (17)	1	41/M	NPS	Neg	Yes	GBS	IVIG, AZ, chloroquine	Non-fatal
Bigaut et al. (18)	2	43/M	NPS	Neg	Yes	GBS	IVIG	Non-fatal
		70/F	NPS	Neg	Yes	GBS	IVIG	Non-fatal
Bodro et al. (19)	2	25/M	NPS	Neg	No	Encephalitis	AC, antibiotics	Non-fatal
		49/M	NPS	Neg	No	Encephalitis	AC, antibiotics	Non-fatal
Canavero et al. (20)	2	25/F	NPS	Neg	Yes	Post-infectious demyelinating myelitis	Steroid	Non-fatal
		69/M	NPS	Neg	NR	Encephalomyelitis	CF, AC, L/R, HCQ, steroid, IVIG	Non-fatal
Casez et al. (21)	1	96/F	Serology	Neg	Yes	Encephalitis	NR	NR
Cebrián et al. (22)	1	74/F	NPS	Pos	No	Headache	NSAIDs, CF, HCQ, L/R	Non-fatal
Chakraborty et al. (23)	1	59/F	NPS, OPS	Neg	No	Acute transverse myelitis	Steroids and antipyretics	Fatal
Chan et al. (24)	1	58/M	OPS	Neg	No	GBS	IVIG	Non-fatal
Chauffier et al. (25)	1	47/M	NPS	Neg	No	Encephalopathy	No medical treatment	Non-fatal
Chaumont et al. (26)	1	69/M	BAL	Neg	Yes	Meningoencephalitis	AC, HCQ, AZ	Non-fatal
Chow et al. (27)	1	60/M	NPS	Neg	Yes	Acute transverse myelitis	Steroid	Non-fatal
Civardi et al. (28)	1	72/F	NPS	Neg	Yes	GBS	IVIG, HCQ, Doxycycline	Non-fatal
Cohen et al. (29)	1	45/M	NPS	Neg	Yes	Parkinson's disease	Steroid, biperiden	Non-fatal
Corrêa et al. (30)	1	51/F	NS	Neg	Yes	Enecephalomyeloradiculitis	Steroid, PE, azathioprine	Non-fatal
De Gennaro et al. (31)	2	42/M	NPS	Neg	No	Cranial neuritis	Remdesivir, sedatives, curare, IVIG	Non-fatal
		67/M	NPS	Neg	No	Cranial neuritis	Antibiotics, anesthetic, noradrenalin, IVIG	Non-fatal
Demirci Otluoglu et al. (32)	1	48/M	CSF	Pos	Yes	Encephalomyelitis	HCQ, Favipiravir, P/T,levetiracetam, steroid, AC	Non-fatal
Dijkstra et al. (33)	1	44/M	NPS	Neg	Yes	Myoclonic syndrome	Steroid and IVIG	Non-fatal
Fadakar et al. (34)	1	47/M	NPS, OPS	Pos	No	Cerebellitis	L/R	Non-fatal
Grimaldi et al. (35)	1	72/M	NPS	Neg	No	Encephalitis	IVIG, steroid, benzodiazepines	Non-fatal
Gutiérrez-Ortiz et al. (36)	2	50/M	OPS	Neg	Yes	Miller fisher syndrome	IVIG	Non-fatal
		39/M	OPS	Neg	NR	PNC	Acetaminophen	Non-fatal
Helbok et al. (37)	1	68/M	Serology	Neg	Yes	GBS	Steroid, IVIG, PE	Non-fatal
Huber et al. (38)	1	21/F	Serology	Neg	Yes	Myasthenia gravis	IVIG and pyridostigmine	Non-fatal
Le Guennec et al. (39)	1	69/M	TA	Neg	Yes	Status epilepticus	Levetiracetam and IVIG	Non-fatal
Lim et al. (40)	1	55/F	NPS	Neg	Yes	Psychotic disorder	Benzodiazepine, antipsychotic	Non-fatal
Moore et al. (41)	1	28/M	NPS	Neg	Yes	Multiple sclerosis	Steroid	Non-fatal
Muccioli et al. (42)	1	47/F	NPS	Neg	Yes	Encephalopathy	Tocilizumab	Non-fatal
Naddaf et al. (43)	1	58/F	Serology	Neg	No	GBS	HCQ, zinc, steroid, PE	Non-fatal
Novi et al. (44)	1	64/F	CSF	Pos	Yes	ADEM	Steroid with OPT, IVIG	Non-fatal
Oguz-Akarsu et al. (45)	1	53/F	NPS	Neg	No	GBS	PE, HCQ, AZ	Non-fatal
Palao et al. (46)	1	29/F	Serology	Neg	Yes	Multiple sclerosis	Steroid with OPT	Non-fatal
Pascual-Goñi et al. (47)	2	60/F	NPS	Neg	Yes	Encephalopathy	Thiamine, pyridoxine, HCQ, AZ	Non-fatal
		35/F	NPS	Neg	NR	Encephalopathy	Thiamine and pyridoxine	Non-fatal
Riva et al. (48)	1	^NR^/M	Serology	Neg	Yes	GBS	IVIG	Non-fatal
Umapathi et al. (49)	2	59/M	NPS	Neg	No	ADEM	Low molecular weight heparin, IVIG	Non-fatal
		73/M	NPS	Neg	NR	Encephalopathy	Interferon-beta 1b, L/R, steroid	Fatal
Vandervorst et al. (50)	1	29/M	NPS	Neg	Yes	Encephalitis	HCQ, nebivolol, amlodipine, antipsychotic, benzodiazepines	Non-fatal
Zanin et al. (51)	1	54/F	Pos; swab unclear	Neg	Yes	Brain & spine demyelinating lesions	ART, HCQ, antiepileptics, steroid	Non-fatal
Zhou et al. (52)	1	26/M	NS, OPS	Neg	No	MOG-IgG-MD	Steroid with OPT	Non-fatal
Zoghi et al. (53)	1	21/M	Serology	Neg	No	Central demyelinating brain injury	PE, antibiotics, AC	Non-fatal
**Case series**								
Cao et al. (54)	5	49/M	NPS/TA	Neg	NR	Encephalitis	Steroid and PE	Non-fatal
		56/M	NPS/TA	Neg	NR	Encephalitis	Steroid and PE	Non-fatal
		61/M	NPS/TA	Neg	NR	Encephalitis	Steroid and PE	Non-fatal
		37/M	NPS/TA	Neg	NR	Encephalitis	Steroid and PE	Fatal
		77/F	NPS/TA	Neg	Yes	Encephalitis	Steroid and PE	Fatal
Delorme et al. (55)	4	72/M	NPS	Neg	Yes	Encephalopathy	IVIG	Non-fatal
		66/F	NPS	Neg	NR	Encephalopathy	IVIG and steroid	Non-fatal
		60/F	NPS	Neg	NR	Encephalopathy	Steroid, antidepressants	Non-fatal
		69/M	NPS	Neg	Yes	Encephalopathy	Levetiracetam, sedative, IVIG, steroid	Non-fatal
Manganotti et al. (56)	4	72/M	NPS	Neg	Yes	GBS	HCQ, antivirals, steroid, tocilizumab	Non-fatal
		72/M	NPS	Neg	Yes	GBS	HCQ, L/R, steroid	Non-fatal
		49/F	NPS	Neg	Yes	GBS	HCQ, L/R, steroid	Non-fatal
		76/M	NPS	Neg	Yes	GBS	HCQ, antivirals, steroid, tocilizumab, antibiotics, fluconazole	Non-fatal
Neumann et al. (57)	30	81/M	NPS	Neg	NR	TIA	NR	NR
		25/F	NPS	Neg	NR	CVST	NR	NR
		48/F	BAL	Neg	NR	Encephalitis-HSV-1	NR	NR
		73/F	NPS	Neg	NR	Suspected post-stroke movement disorder	NR	NR
		63/M	BAL	Neg	NR	Miller fisher syndrome	NR	NR
		58/M	BAL	Neg	NR	Encephalopathy with Seizure	NR	NR
		75/F	NPS	Neg	Yes	Encephalopathy DD limbic Encephalitis	NR	NR
		66/M	NPS, BAL	Neg	NR	Intracranial hemorrhage	NR	NR
		56/M	OPS, BAL	Neg	NR	Encephalopathy, CIP	NR	NR
		41/F	OPS	Neg	NR	Osmotic demyelination syndrome	NR	NR
		68/M	BAL	Neg	NR	Seizure	NR	NR
		64/M	OPS, BAL	Neg	NR	Encephalopathy, CIP	NR	NR
		57/M	OPS, BAL	Neg	NR	Status epilepticus	NR	NR
		75/M	OPS, BAL	Neg	NR	Encephalopathy, CIP	NR	NR
		47/M	OPS, BAL	Neg	NR	Encephalopathy, CIP	NR	NR
		50/M	OPS, BAL	Neg	NR	Seizure	NR	NR
		51/M	OPS, BAL	Neg	NR	Encephalopathy	NR	NR
		65/F	OPS	Neg	NR	Encephalopathy	NR	NR
		45/M	OPS	Neg	NR	Unclear headache	NR	NR
		68/F	OPS	Neg	NR	Encephalopathy	NR	NR
		81/M	OPS, BAL	Neg	NR	Encephalopathy	NR	NR
		48/M	OPS	Neg	Yes	UVN	NR	NR
		58/F	OPS	Neg	NR	UANP	NR	NR
		80/M	OPS	Neg	Yes	Encephalopathy	NR	NR
		70/M	OPS, BAL	Neg	NR	CIP, Ischemic stroke	NR	NR
		76/F	OPS, BAL	Neg	NR	Prolonged coma	NR	NR
		79/F	OPS, BAL	Neg	NR	GBS and encephalopathy	NR	NR
		28/F	OPS	Neg	NR	Ischemic stroke	NR	NR
		68/M	OPS	Neg	NR	Seizures	NR	NR
		86/F	OPS	Neg	NR	GBS	NR	NR
Perrin et al. (58)	5	71/F	NPS	Neg	NR	Encephalopathy	Levetiracetam and steroid	Fatal
		64/M	NPS	Neg	NR	Encephalopathy	L/R, benzodiazepine, steroid, IVIG	Non-fatal
		53/F	NPS	Neg	NR	Encephalopathy	HCQ	Non-fatal
		51/M	NPS	Neg	NR	Encephalopathy	HCQ	Non-fatal
		67/M	NPS	Neg	Yes	Encephalopathy	HCQ, steroids	Non-fatal
Toscano et al. (59)	5	77/F	NPS	Neg	NR	GBS	IVIG	Non-fatal
		23/M	NPS	Neg	NR	GBS	IVIG	Non-fatal
		55/M	NPS	Neg	NR	GBS	IVIG	Non-fatal
		76/M	NPS	Neg	Yes	GBS	IVIG	Non-fatal
		61/M	Serology	Neg	Yes	GBS	IVIG and PE	Non-fatal

*AC, acyclovir; ADEM, Acute disseminating encephalomyelitis; ART, antiretroviral therapy; AZ, azithromycin; BAL, Bronchoalveolar lavage; CF, ceftriaxone; CIP, critical illness polyneuropathy; CSF, cerebrospinal fluid; CVST, Cerebral venous sinus thrombosis; DD, differential diagnosis; GBS, Guillain-Barré syndrome; HCQ, hydroxychloroquine; HSV, herpes simplex virus; IVIG, intravenous immunoglobulin; LOS, loss of smell; L/R, lopinavir and ritonavir; MOG-IgG-MD, myelin oligodendrocyte glycoprotein- antibody-mediated disease; NPS, nasopharyngeal swab; NS, nasal swab; NSAIDs, Non-steroidal anti-inflammatory drug; OPS, oropharyngeal swab; OPT, oral prednisolone tapering; PCR, polymerase chain reaction; PE, plasma exchange; PNC, Polyneuritis cranialis; P/T, piperacillin and tazobactam; SARS-CoV-2, severe acute respiratory syndrome coronavirus-2; TA, tracheal aspirate; TIA, Transient ischemic attack; UVN, Unilateral Vestibular Neuritis*.

*NR denotes data not reported in the studies, F means female, M means male, Pos means positive, and Neg means negative*.

### Neurological Aspects of COVID-19

#### Clinical Presentation

The neurological symptoms observed in COVID-19 individuals were diverse. The most common COVID-19-related neurological symptoms were smell disorder, taste disorder, headache, myalgia, altered consciousness, related paresis, and related cognitive and behavioral disturbances.

#### Neurological Diagnosis

The neurological diagnosis made after the neurological and radiological examination was localized to CNS (61.39%), peripheral nervous system (32.67%), or both (5.94%). In a study comprising 30 participants, six patients were diagnosed with two neurological disorders (57). The most common neurological diagnosis included Guillain-Barré syndrome and its variant (24%), followed by encephalopathy (21%) (Table 1).

#### Treatment and Outcomes

Information on the therapeutic management of COVID-19 was available for only 69 patients, including one patient who did not require medical treatment. In most cases, therapeutic management of COVID-19 patients involved combinational therapies. Common treatments included steroids administration (*n* = 32/69), intravenous immunoglobulin infusion (*n* = 28/69), hydroxychloroquine (*n* = 18/69), and plasma exchange (*n* = 11/69). Other medications used in the management of COVID-19 patients are shown in Table 1. The administration of medications resulted in neurological improvement in most patients. There were 63 non-fatal cases, five fatal cases, and one patient did not regain consciousness even after sedation was discontinued.

#### SARS-CoV-2 PCR Assay Results

Patients were confirmed to be COVID-19 positive when tested positive in PCR assay from nasopharyngeal swab or nasal swab (50/101), oropharyngeal swab (14/101), bronchoalveolar lavage (5/101), tracheal aspirate (1/101), or a combination of them (20/101). The PCR assay was positive in one study, but the swab used was not specified (51). Two patients were confirmed COVID-19 positive with the presence of SARS-CoV-2 in CSF (32, 44), and in eight patients with negative PCR test, COVID-19 infection was diagnosed with the presence of anti-SARS-CoV-2 in serum (Table 1) (21, 37, 38, 43, 46, 48, 53, 59).

CSF PCR assay for SARS-CoV-2 was positive for only four (3.96%) patients (22, 32, 34, 44) and negative in 97 (96.04%) patients (15–21, 23–31, 33, 35–43, 45–59). Of the 101 patients, information on the status of anosmia was available in 55 patients (51 patients had negative CSF PCR results, while four had positive CSF PCR results). Out of 51 patients with negative CSF PCR results, 38 had smell disorder, while 13 had no nasal symptoms. Meanwhile, two of the four patients with positive CSF PCR results for SARS-CoV-2 had olfactory dysfunction, while the other two did not (Table 1).

## Discussion

This systematic review identified studies that performed CSF PCR assay for SARS-CoV-2 in COVID-19 positive patients and reported anosmia status to identify the common neurological manifestations associated with COVID-19 and to analyze the interrelation between CSF PCR results and anosmia. The neurological manifestations of COVID-19 are diverse. There was an equal number of patients with and without olfactory disorders who had positive CSF PCR results for SARS-CoV-2.

COVID-19 can trigger other autoimmune neurological complications such as neuromyelitis optica spectrum disorders or multiple sclerosis (30, 41, 46), which should be identified and treated promptly (44). In addition, COVID-19 patients with olfactory disorders and other severe neurological symptoms should be examined for possible neurodegenerative disease when suspected of having one (29).

In our study, ~4% of the participants had positive CSF PCR assay for SARS-CoV-2, similar to the finding of one study, which showed positive results in 6% of the participants, indicating SARS-CoV-2 neuroinvasion is a rare occurrence (3). However, negative CSF PCR results for SARS-CoV-2 may be due to delayed immune-mediated neurological damage after viral clearance (51, 53). Furthermore, the sensitivity decreases if samples are tested after a long period of symptom onset, giving negative results (43, 58). In addition, according to our review, only 44% of the published articles on CSF studies performed CSF PCR assay for SARS-CoV-2 in COVID-19 infected patients who experienced neurological symptoms. Therefore, despite the procedural and logistical complexity, the authors suggest an early collection of CSF samples, performing CSF PCR assay for SARS-CoV-2, detecting anti-neuronal autoantibodies, and using 18 F-fluorodeoxyglucose positron emission tomography in suspected cases could aid in the diagnosis and management of the patients, notably in magnetic resonance imaging negative cases (35, 51). Although the additional financial concern associated with the CSF PCR assay cannot be avoided, there were cases of testing positive in CSF PCR assay despite being negative in a nasal PCR or rapid COVID-19 test (32, 44). In addition, cost-effective studies in other neurotropic viruses have shown that the CSF PCR assay is cost-effective; similar studies in COVID-19 are required (60). Furthermore, a negative CSF PCR assay does not rule out the presence of the virus in the CNS; therefore, further studies of SARS-CoV-2 antibodies are required (57). Moreover, a recent study has shown that SARS-CoV-2 retrograde neuroinvasion *via* the olfactory route causes neuroinflammation (9). The detection of SARS-CoV-2 in the olfactory epithelium and various radiological findings in patients with COVID-19 suggests that despite the rarity of SARS-CoV-2 neuroinvasion *via* the olfactory system, it should not be overlooked (9, 21, 39, 61).

**Figure 3:**
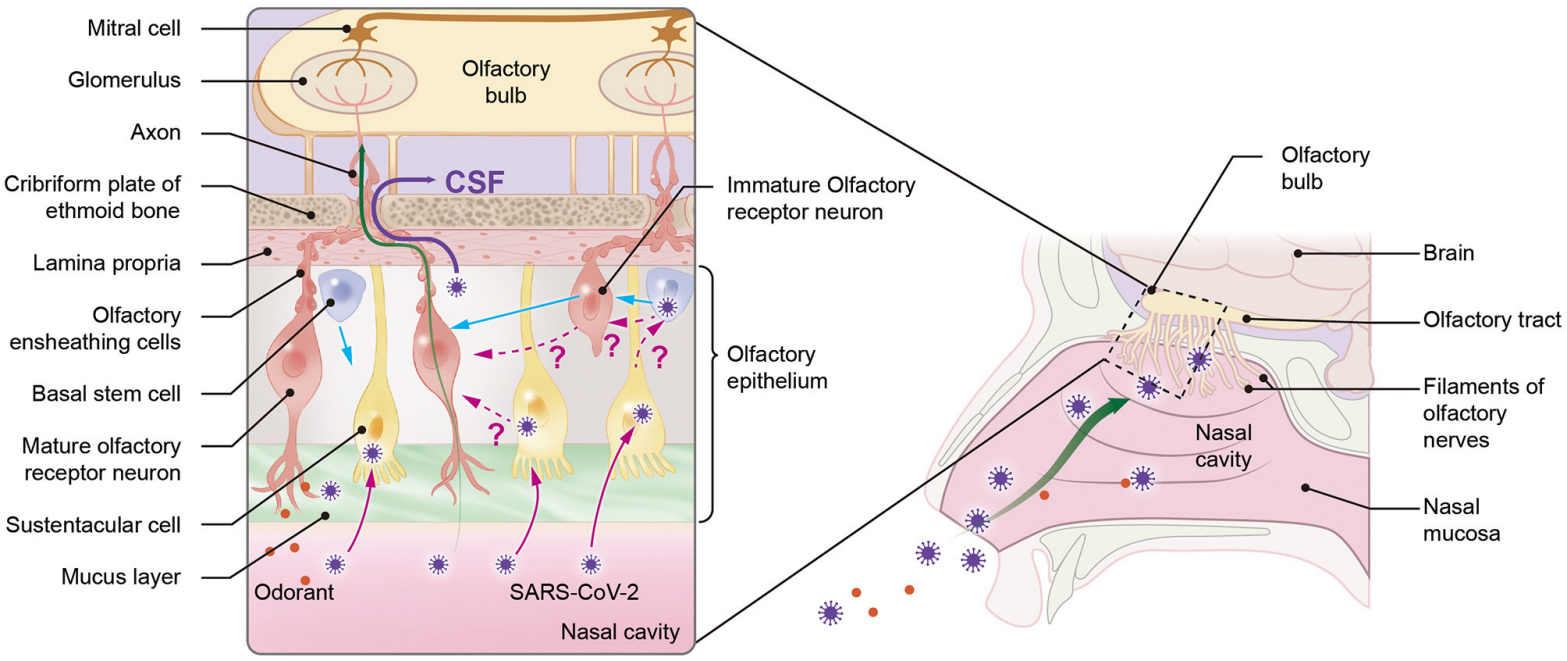
Possible mechanisms of neuroinvasion of severe acute respiratory syndrome coronavirus-2 (SARS-CoV-2) *via* the olfactory pathway. SARS-CoV-2 can enter the central nervous system (CNS) through the olfactory system in one of two ways: directly through the cerebrospinal fluid (CSF) by crossing the channels created by olfactory ensheathing cells (OECs) (purple straight line) or through olfactory receptor neurons (ORNs). Since ORNs lack angiotensin-converting enzyme-2 (ACE-2), viruses are hypothesized to be transferred from sustentacular (SUS) cells, which contain ACE-2, either directly to mature ORNs (mORNs) or to stem cells (also containing ACE-2), which can then transfer the virus to immature ORNs (iORNs) during ORN regeneration process, where infected iORNs could grow into infected mORNs. The pink dotted line represents the hypotheses. Viruses can directly enter the CNS from ORN through ACE-2-independent mechanisms (green straight line) using factors such as neurolipin-1. The blue straight line represents the regeneration of ORNs and SUS cells from stem cells.

Similar to other respiratory neurotropic viruses, the direct neuroinvasion of SARS-CoV-2 in COVID-19 patients could occur mainly in two ways: damage to the olfactory epithelium or diffusion through the olfactory ensheathing cell (OEC) (1, 2) (Figure 3). Although ORNs of humans do not express SARS-CoV-2 entry proteins, factors other than angiotensin-converting enzyme-2 may be involved in a viral entry, such as neurolipin-1, which is highly expressed in ORNs (62–64) or SARS-CoV-2 can have non-neuronal mechanism (6, 9, 63). The neuronal and non-neuronal damage of the olfactory epithelium are responsible for the mechanism of loss of smell observed in COVID-19 patients (6, 7, 9). Nevertheless, viruses that are rapidly transported to the olfactory bulb before being affected by ORN apoptotic actions may invade the CNS (5).

In addition, viruses as small as 100nm can also diffuse *via* the channels formed by OEC gaining direct access to the CSF (1, 65). The size of SARS-CoV-2 ranges from 60 to 140 nm (66). Additionally, direct infection of the OEC can release viruses into these channels and subsequently transport the virus to the olfactory bulb (1). Thus, SARS-CoV-2 with a smaller size can utilize this mode of viral transmission.

Studies analyzing the olfactory mucosa of COVID-19 patients with and without anosmia are required to acknowledge that apoptosis of ORNs is the cause of COVID-19-related anosmia. Future studies with a larger sample size involving nasal brush sampling method and CSF PCR assay can be performed on COVID-19 patients to determine whether apoptosis of ORNs could provide neuroprotection in COVID-19 patients with anosmia (9).

This study has several limitations. Olfactory mucosa biopsy is required to effectively analyze the association between apoptosis of ORNs with anosmia and neuroprotection. However, few studies were included in this analysis. Because the biopsy is an invasive procedure, it is rarely done in patients with COVID-19 only for research purposes (9), unlike animal studies. Additionally, studies that determine whether apoptosis of ORNs occurs in COVID-19 patients experiencing anosmia and SARS-CoV-2 CSF PCR assays are not available. For these reasons, the study design for analyzing the hypothesis was only feasible with observational studies. Though the risk of bias assessment showed an overall low risk, fundamental bias from the study design cannot be fully excluded. The findings from this review are not directly comparable with the results from other neurotropic viruses till these unanswered issues are solved. The number of patients with positive CSF PCR results did not differ by anosmia status, which may be related to the limited sample size and non-standard CSF PCR assay procedures. The CSF PCR assay is not commonly performed in COVID-19 patients with neurological manifestation. In this study, among COVID-19 patients with neurological manifestation, only 44% of patients underwent PCR assay for SARS-CoV-2 in the CSF to identify COVID-19 related neurological disorders. Though anosmia is common in COVID-19 patients, underreporting issues cannot be ignored, and because of limitation to our methodology, the neurological manifestations observed in individuals with COVID-19 cannot be generalized. Similarly, the possibility of an indirect mechanism of neuroinvasion of SARS-CoV-2 should not be overlooked. We could not investigate the neurological aspects of different strains of SARS-CoV-2 in COVID-19 infected patients and geographical and temporal relationships, particularly those concerning olfactory alteration, because information about the SARS-CoV-2 strain along with geographical and temporal information was not available in the included studies. Future studies with proper sample sizes involving definitive methods such as the nasal mucosa sampling method could provide a clear answer to the association between apoptosis of ORNs with anosmia and neuroprotection.

## Conclusion

The neurological spectrum of COVID-19 is wide. Direct neuroinvasion of SARS-CoV-2 *via* the olfactory route is uncommon. Although previous experimental models of respiratory neurotropic viruses have demonstrated that apoptosis of the olfactory nerve blocks its neuroinvasive ability, this remains controversial in the case of SARS-CoV-2, since at present, human evidence is too scare limiting any conclusion to be drawn about the protection role of virus' olfactory mucosa invasion toward CNS invasion. More research with definitive methods is required to study the neuroprotective potential of ORN apoptosis in COVID-19 patients.

## Data Availability Statement

The original contributions presented in the study are included in the article/Supplementary Material, further inquiries can be directed to the corresponding author/s.

## Author Contributions

EK had full access to all the data in the study and took responsibility for the integrity of the data and the accuracy of the data analysis. SP and EK: conceptualization, writing original draft, and formal analysis. SP, SO, and EK: data acquisition and writing review and editing. EK: funding and supervision. All authors contributed to the article and approved the submitted version.

## Funding

This work was funded by a grant from the Korean government, South Korea (Ministry of Science and ICT, MICT; NRF-2021R1F1A1062044) and by the Basic Science Research Program through the National Research Foundation of Korea funded by the Ministry of Education, South Korea (Grant Number 2021R1A6A1A03044296). The funder had no role in the trial design, data collection, data interpretation, or report preparation.

## Conflict of Interest

The authors declare that the research was conducted in the absence of any commercial or financial relationships that could be construed as a potential conflict of interest.

## Publisher's Note

All claims expressed in this article are solely those of the authors and do not necessarily represent those of their affiliated organizations, or those of the publisher, the editors and the reviewers. Any product that may be evaluated in this article, or claim that may be made by its manufacturer, is not guaranteed or endorsed by the publisher.
