# Supplementary material for: A Systematic Review on Neurological Aspects of COVID-19: Exploring the Relationship Between COVID-19-Related Olfactory Dysfunction and Neuroinvasion
Source: Front Neurol. 2022 Jul 15;13:887164. doi: 10.3389/fneur.2022.887164 (PMC9334857; doi:10.3389/fneur.2022.887164)
Supplement: Supplementary file 1 [file Data_Sheet_1.docx]

**S1 Table.** Quality Assessment for Included Studies with the JBI checklist for Case Reports

| **Study** | **Demographics described** | **Patient’s history described** | **Current clinical condition described** | **Diagnostic test described** | **Treatment clearly described** | **Post-intervention condition described** | **Adverse events identified** | **Takeaway lessons** | **% Yes** | **Risk** |
| --- | --- | --- | --- | --- | --- | --- | --- | --- | --- | --- |
| Andriuta et al. [15] | ? | ✓ | ✓ | ✓ | ✕ | ✕ | ✕ | ? | 37.5% | High |
| Assini et al. [16] | ✓ | ✕ | ✓ | ✓ | ✓ | ✓ | ✕ | ✓ | 75% | Low |
| Atakla et al. [17] | ✓ | ✕ | ✓ | ✓ | ✓ | ✓ | ✓ | ✓ | 87% | Low |
| Bigaut et al. [18] | ✓ | ? | ✓ | ✓ | ✓ | ✓ | ✕ | ✓ | 75% | Low |
| Bodro et al. [19] | ✓ | ✓ | ✓ | ✓ | ✓ | ✓ | ✕ | ✓ | 87% | Low |
| Canavero et al. [20] | ✓ | ? | ✓ | ✓ | ✓ | ✓ | ✕ | ✓ | 75% | Low |
| Casez et al. [21] | ✓ | ✕ | ✓ | ✓ | ✕ | ✕ | ✕ | ✓ | 50% | Moderate |
| Cebrián et al. [22] | ✓ | ✓ | ✓ | ✓ | ✓ | ✓ | ✕ | ✓ | 87% | Low |
| Chakraborty et al. [23] | ✓ | ✓ | ✓ | ✓ | ✓ | ✓ | ✕ | ✓ | 87% | Low |
| Chan et al. [24] | ✓ | ✓ | ✓ | ✓ | ✓ | ✓ | ✕ | ✓ | 87% | Low |
| Chauffier et al. [25] | ✓ | ✓ | ✓ | ✓ | ✓ | ✓ | ✕ | ✓ | 87% | Low |
| Chaumont et al. [26] | ✓ | ✓ | ✓ | ✓ | ✓ | ✓ | ✕ | ✓ | 87% | Low |
| Chow et al. [27] | ✓ | ✓ | ✓ | ✓ | ✓ | ✓ | ✕ | ✓ | 87% | Low |
| Civardi et al. [28] | ✓ | ✕ | ✓ | ✓ | ✓ | ✓ | ✕ | ✓ | 75% | Low |
| Cohen et al. [29] | ✓ | ✓ | ✓ | ✓ | ✓ | ✓ | ? | ✓ | 87% | Low |
| Corrêa et al. [30] | ✓ | ✓ | ✓ | ✓ | ✓ | ✓ | ✕ | ✓ | 87% | Low |
| De Gennaro et al. [31] | ✓ | ✓ | ✓ | ✓ | ✓ | ✓ | ✕ | ✓ | 87% | Low |
| Demirci Otluoglu et al. [32] | ✓ | ✓ | ✓ | ✓ | ✓ | ✓ | ✕ | ✓ | 87% | Low |
| Dijkstra et al. [33] | ✓ | ✓ | ✓ | ✓ | ✓ | ✓ | ✕ | ✓ | 87% | Low |
| Fadakar et al. [34] | ✓ | ✓ | ✓ | ✓ | ✓ | ✓ | ✕ | ✓ | 87% | Low |
| Grimaldi et al. [35] | ✓ | ✓ | ✓ | ✓ | ✓ | ✓ | ✓ | ✓ | 100% | Low |
| Gutiérrez-Ortiz et al. [36] | ✓ | ✓ | ✓ | ✓ | ✓ | ✓ | ✕ | ✓ | 87% | Low |
| Helbok et al. [37] | ✓ | ✓ | ✓ | ✓ | ✓ | ✓ | ? | ✓ | 87% | Low |
| Huber et al. [38] | ✓ | ✓ | ✓ | ✓ | ✓ | ✓ | ✕ | ✓ | 87% | Low |
| **S1 Table (continue)** | | | | | | | | | | |
| **Study** | **Demographics described** | **Patient’s history described** | **Current clinical condition described** | **Diagnostic test described** | **Treatment clearly described** | **Post-intervention condition described** | **Adverse events identified** | **Takeaway lessons** | **% Yes** | **Risk** |
| Le Guennec et al. [39] | ✓ | ✓ | ✓ | ✓ | ✓ | ✓ | ✕ | ✓ | 87% | Low |
| Lim et al. [40] | ✓ | ✓ | ✓ | ✓ | ✓ | ✓ | ? | ✓ | 87% | Low |
| Moore et al. [41] | ✓ | ✓ | ✓ | ✓ | ✓ | ✓ | ✕ | ✓ | 87% | Low |
| Muccioli et al. [42] | ✓ | ✓ | ✓ | ✓ | ✓ | ✓ | ✕ | ✓ | 87% | Low |
| Naddaf et al. [43] | ✓ | ✕ | ✓ | ✓ | ✓ | ✓ | ✕ | ✓ | 75% | Low |
| Novi et al. [44] | ✓ | ✓ | ✓ | ✓ | ✓ | ✓ | ✕ | ✓ | 87% | Low |
| Oguz-Akarsu et al. [45] | ✓ | ✓ | ✓ | ✓ | ✓ | ✓ | ✕ | ✓ | 87% | Low |
| Palao et al. [46] | ✓ | ✓ | ✓ | ✓ | ✓ | ✓ | ✕ | ✓ | 87% | Low |
| Pascual-Goñi et al. [47] | ✓ | ? | ✓ | ✓ | ✓ | ✓ | ✕ | ✓ | 75% | Low |
| Riva et al. [48] | ? | ✓ | ✓ | ✓ | ✓ | ✓ | ✕ | ✓ | 75% | Low |
| Umapathi et al. [49] | ✓ | ? | ✓ | ✓ | ✓ | ✓ | ✕ | ✓ | 75% | Low |
| Vandervorst et al. [50] | ✓ | ✓ | ✓ | ✓ | ✓ | ✓ | ✕ | ✓ | 87% | Low |
| Zanin et al. [51] | ✓ | ✓ | ✓ | ✓ | ✓ | ✓ | ✕ | ✓ | 87% | Low |
| Zhou et al. [52] | ✓ | ✓ | ✓ | ✓ | ✓ | ✓ | ✕ | ✓ | 87% | Low |
| Zoghi et al. [53] | ✓ | ✓ | ✓ | ✓ | ✓ | ✓ | ✕ | ✓ | 87% | Low |

**Abbreviations:** JBI Joanna Briggs Institute **Note:** ‘✓’ indicates yes, ‘✕’ indicates no and ‘?’ indicates unclear.

The risk of bias was ranked as high when the study reached up to 49% of “yes” scores, moderate when the study reached from 50 to 69% of “yes” scores, and low when the study reached more than 70% of “yes” scores.^1^

1. Melo G, Dutra KL, Rodrigues Filho R, et al. Association between psychotropic medications and presence of sleep bruxism: A systematic review. *J Oral Rehabil* 2018;45(7):545-54. doi: 10.1111/joor.12633

**S2 Table.** Quality Assessment for Included Studies with the JBI checklist for Case Series

| **Study** | **Clear inclusion criteria** | **Standard and reliable measurement of condition** | **Valid methods for condition identification** | **Consecutive inclusion of participants** | **Complete inclusion of participants** | **Clear reporting of demographics** | **Clear reporting of clinical information** | **Clear reporting of outcomes or follow-up results** | **Clear reporting of geographic regions and population** | **Appropriate statistical analysis** | **% Yes** | **Risk** |
| --- | --- | --- | --- | --- | --- | --- | --- | --- | --- | --- | --- | --- |
| Cao et al. [54] | ✓ | ✓ | ✓ | ✓ | ✕ | ✓ | ✓ | ✓ | ? | N/A | 70% | Low |
| Delorme et al. [55] | ✓ | ✓ | ✓ | ✓ | ✓ | ✓ | ✓ | ✓ | ? | N/A | 80% | Low |
| Manganotti et al. [56] | ✓ | ✓ | ✓ | ✓ | ? | ✓ | ✓ | ✓ | ? | N/A | 70% | Low |
| Neumann et al. [57] | ✓ | ✓ | ✓ | ✓ | ? | ✓ | ✓ | ✕ | ? | N/A | 60% | Moderate |
| Perrin et al. [58] | ✓ | ✓ | ✓ | ✓ | ✕ | ✓ | ✓ | ✓ | ? | N/A | 70% | Low |
| Toscano et al. [59] | ✓ | ✓ | ✓ | ✓ | ? | ✓ | ✓ | ✓ | ? | N/A | 70% | Low |

Abbreviations: JBI Joanna Briggs Institute

Note ‘✓’ indicates yes, ‘✕’ indicates no, ‘?’ indicates unclear and N/A indicates not applicable

The risk of bias was ranked as high when the study reached up to 49% of “yes” scores, moderate when the study reached from 50 to 69% of “yes” scores, and low when the study reached more than 70% of “yes” scores.^1^

1. Melo G, Dutra KL, Rodrigues Filho R, et al. Association between psychotropic medications and presence of sleep bruxism: A systematic review. *J Oral Rehabil* 2018;45(7):545-54. doi: 10.1111/joor.12633
